# FPGA implementation of a biological neural network based on the Hodgkin-Huxley neuron model

**DOI:** 10.3389/fnins.2014.00379

**Published:** 2014-11-21

**Authors:** Safa Yaghini Bonabi, Hassan Asgharian, Saeed Safari, Majid Nili Ahmadabadi

**Affiliations:** ^1^Cognitive Robotic Lab., School of Electrical and Computer Engineering, College of Engineering, University of TehranTehran, Iran; ^2^Research Center of Information Technology, Department of Computer Engineering, Iran University of Science and TechnologyTehran, Iran; ^3^High Performance Embedded Computing Lab., School of Electrical and Computer Engineering, College of Engineering, University of TehranTehran, Iran; ^4^School of Cognitive Sciences, Institute for Research in Fundamental Sciences, IPMTehran, Iran

**Keywords:** Hodgkin-Huxley, neural pool, neural network, digital hardware implementation, FPGA

## Abstract

A set of techniques for efficient implementation of Hodgkin-Huxley-based (H-H) model of a neural network on FPGA (Field Programmable Gate Array) is presented. The central implementation challenge is H-H model complexity that puts limits on the network size and on the execution speed. However, basics of the original model cannot be compromised when effect of synaptic specifications on the network behavior is the subject of study. To solve the problem, we used computational techniques such as CORDIC (Coordinate Rotation Digital Computer) algorithm and step-by-step integration in the implementation of arithmetic circuits. In addition, we employed different techniques such as sharing resources to preserve the details of model as well as increasing the network size in addition to keeping the network execution speed close to real time while having high precision. Implementation of a two mini-columns network with 120/30 excitatory/inhibitory neurons is provided to investigate the characteristic of our method in practice. The implementation techniques provide an opportunity to construct large FPGA-based network models to investigate the effect of different neurophysiological mechanisms, like voltage-gated channels and synaptic activities, on the behavior of a neural network in an appropriate execution time. Additional to inherent properties of FPGA, like parallelism and re-configurability, our approach makes the FPGA-based system a proper candidate for study on neural control of cognitive robots and systems as well.

## Introduction

Developing computational tools for simulating the brain networks is of a very special interest, because the models provide powerful means for investigating different characteristics of the neural system. For example, they can be used to find the effect of malfunctioning voltage-gated channels on network level behaviors in specific brain diseases or are employed to track effects of learning on synaptic efficacies and neural behavior. Using the computational tools before undertaking biological experiments can also give some insights into the results of experiments. In addition, computational models can be used as controllers for cognitive robots.

Neurons and neural pools are basis of computational models. Neurons receive sensory signals, process the information, excite/inhibit each other through a complex electrochemical process (Kandel et al., 2000). A neural pool is a group of neurons sharing excitatory or inhibitory property. Neural pools can inhibit or excite each other by means of output signals. Therefore, activity of each neuron can affect the behavior of its pool and other pools in the brain, so specific behavior of the neural networks emerge from interaction of neurons and neural pools (Buzsáki, 2004).

A neural network can be implemented on software or hardware. Due to the sequential execution of software, the parallel nature of neural networks is affected which leads to reduction of execution speed. Implementing neurons on hardware can provide several benefits; including high-speed modeling of big neural networks and preparing responses in real-time, etc. (Indiveri et al., 2011). In the hardware implementation techniques, digital implementations are more preferred vs. analog implementations, based on some of the digital advantages such as noise-robustness, more flexibility, simple real-world interfaces, and easier testability (Muthuramalingam et al., 2008). In addition, digital implementations are more cost-effective and less time consuming (Gatet et al., 2009). One of the other benefits of the digital implementations is their capability for fast development (Indiveri et al., 2011). There are different methods for digital implementation such as ASIC (Application Specific Integrated Circuit), DSP (Digital Signal Processing), and FPGA (Field Programmable Gate Array). DSP-based implementations are not suitable for modeling the parallel behavior of the neurons because of their sequential nature. ASIC implementation is more efficient than FPGA in terms of power and area, but it suffers from lack of re-configurability (Wanhammar, 1999). FPGA has some benefits over DSP and ASIC that we are interested in: it is reconfigurable, so it is useful for rapid prototyping of neural networks (Wang et al., 2014), and it has parallel processing architecture. Therefore, FPGA could be an appropriate solution for hardware implementation of neural networks (Muthuramalingam et al., 2008).

FPGAs have limited usable area and design tool chains, which create difficulty in implementation of large neural networks. Therefore, designs should be optimized in size to be implemented on an FPGA with limited number of resources. In this paper, we design and implement a biologically plausible neural network on an FPGA. There are different biological neuron models; however, we opt for Hodgkin-Huxley (H-H) neural model because of its biological plausibility and inclusion of synaptic details. Due to the FPGA area limitation, we use the reduced order of the previous implementation (Bonabi et al., 2012a). The implemented neural network in this work is a modified model used in Moldakarimov et al. (2005). The implemented network is the basic component of many neural networks; it has two mini-columns, each has two neural pools: an excitatory pool with 60 neurons and an inhibitory pool with 15 neurons. The reason that we are interested in this model is that this model can be used to investigate the competition between neural pools in the brain (Bakhtiari et al., 2012). Using the hardware as an accelerator for reducing the computation time of such neural networks is our main objective. The basis of single pool implementation are stated in Bonabi et al. (2012b). We use MATLAB simulations for high-level design of neural network and the results of simulations are used as a golden model to check correctness of our implementation.

In Materials and Methods, we introduce the model at the neural and the network levels. FPGA implementation of the network is introduced in this section, too. In Results, the validation processes and the implementation results for a set of variables are given. In addition, the hardware system and the speed of processing are discussed. Discussions and Conclusions are given in the last section.

## Materials and methods

### Neuron model

**Figure 1:**
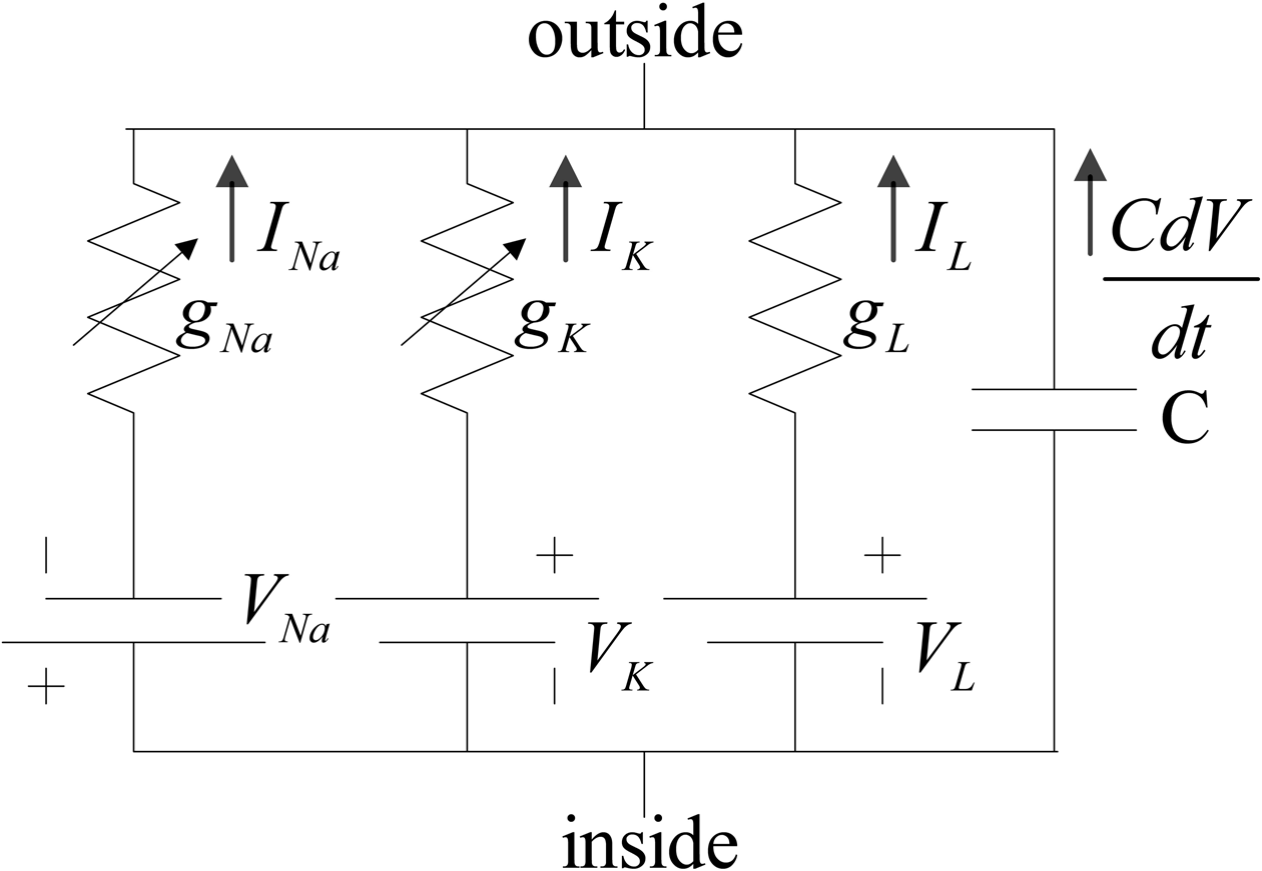
**Hodgkin and Huxley proposed circuit for squid giant axon**. g_K_ and g_Na_, are voltage-dependent conductance (Hodgkin and Huxley, 1952).

The neuron model used in this implementation is the reduced version of the model introduced in Traub and Miles (1991). Ermentrout and Kopell (1998) and Börgers et al. (2005) also used the reduced model to investigate the dynamical behaviors of neural networks. The structure of the model for both of the inhibitory and excitatory neurons is the same. The membrane potential of the neurons follows the H-H equations (Hodgkin and Huxley, 1952). The ionic current that is mainly composed of sodium and potassium ions controls the membrane voltage. Moreover, this voltage regularizes the current flow of the ions by means of voltage-dependent ion channels. There are other ionic currents such as chloride, which their gating variables are independent of the membrane voltage. These ions constitute the leak current. Inside and outside of the membrane do not have equal concentration of ions, which results in an electrical potential. Both concentration gradient and electrical potential controls the current flow. The H-H model is shown in Figure 1.

According to Börgers et al. (2005); Izhikevich (2007) the relationship between input current, membrane voltage, and the complete set of H-H equations come in (1a–4c):

(1a) $$\frac{CdV}{dt}=I-I_{Na}-I_{K}-I_{L}$$

(1b) $$I_{Na}=\bar{g_{Na}}m^{3}h\left(V-V_{Na}\right)$$

(1c) $$I_{K}=\bar{g_{K}}n^{4}\left(V-V_{K}\right)$$

(1d) $$I_{L}=g_{L}\left(V-V_{L}\right)$$

Where,

(2a) $$m=m_{\infty }\left(V\right)=\frac{\alpha_{m}\left(V\right)}{\left[\alpha_{m}\left(V\right)+\beta_{m}\left(V\right)\right]}$$

(2b) $$\alpha_{m}\left(V\right)=\frac{0.32\left(V+54\right)}{1-exp\left[-0.25\left(V+54\right)\right]}$$

(2c) $$\beta_{m}\left(V\right)=\frac{0.28\left(V+27\right)}{exp\left[0.2\left(V+27\right)\right]-1}$$

(3) h=max(1−1.25n, 0)

(4a) $$\frac{dn}{dt}=0.7\left(\alpha_{n}\left(V\right)\left(1-n\right)-\beta_{n}\left(V\right)n\right)$$

(4b) $$\alpha_{n}\left(V\right)=\frac{0.01\left(V+34\right)}{1-exp\left[-0.1\left(V+34\right)\right]}$$

(4c) $$\beta_{n}\left(V\right)=0.125exp\left[-0.0125\left(V+44\right)\right]$$

The typical values and units of the parameters that used in the above equations according to Börgers et al. (2005) are as follows: *C* = 1 μ*F*/*cm*^2^, *g*_*Na*_ = 100 *mS*/*cm*^2^, *V*_*Na*_ = 50 *mV*, *g*_*K*_ = 80 *mS*/*cm*^2^, *V*_*K*_ = −100 *mV*, *g*_*L*_ = 0.1 *mS*/*cm*^2^, *V*_*L*_ = −67 *mV*.

The units of the letters *V*, *t*, and *I* are *mV*, *ms*, and μA/cm^2^ respectively.

### Neural network model

**Figure 2:**
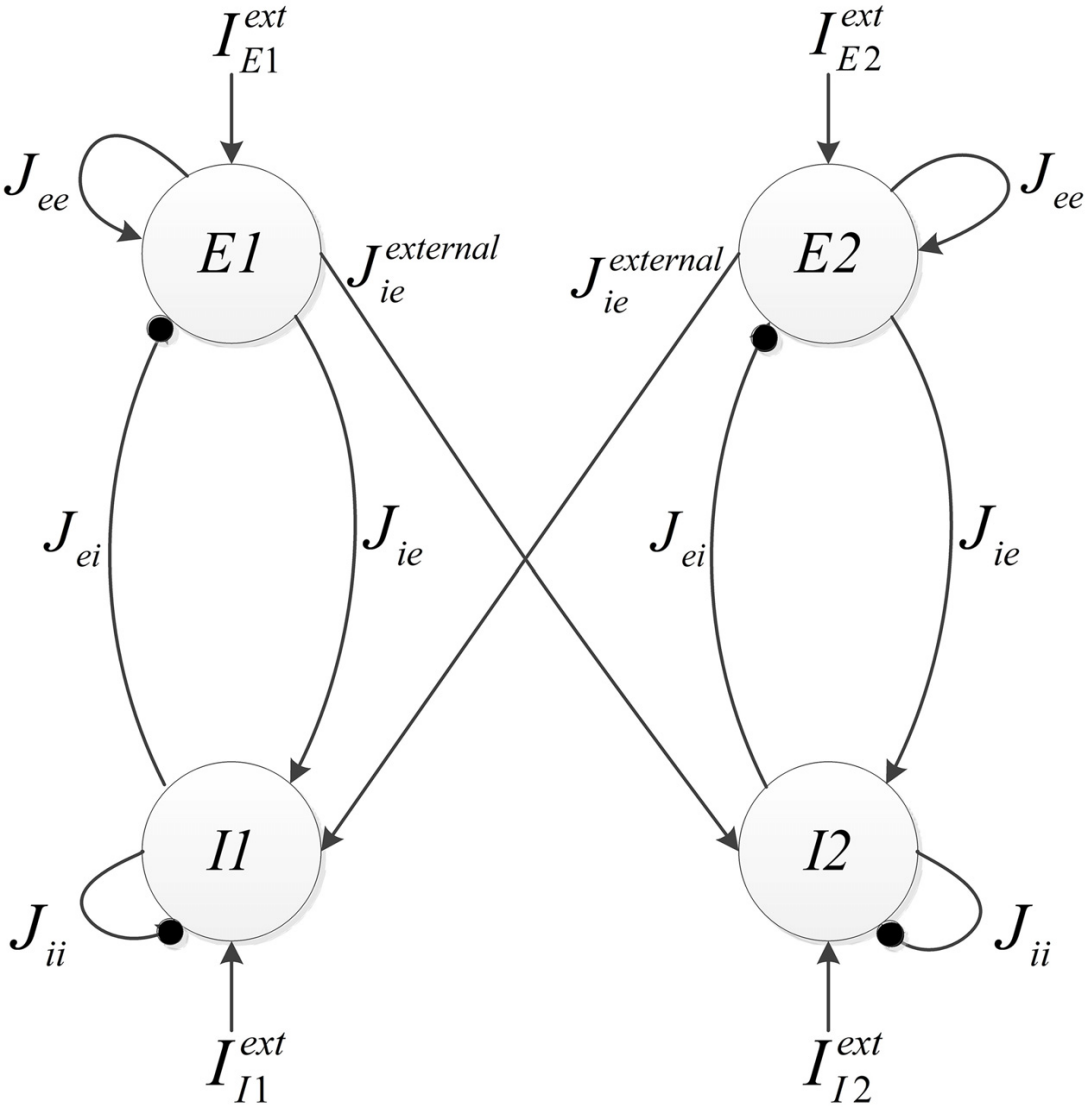
**The structure of neural network, consist of two competitive mini-columns**.

The implemented neural network is composed of two similar mini-columns. Figure 2 shows the network model. Each mini-column has an excitatory and an inhibitory pool. All of the neurons in each pool are connected to each other. By means of specialized structures called synapses, the information is transmitted among neurons. According to Börgers et al. (2005) neurons in each pool are related to each other with neurotransmitter, AMPA (E → E and E → I) and GABA_A_ receptors (I → I and I → E). In order to create a relationship between the two mini-columns, an excitatory synaptic current is given to the inhibitory pool in the adjacent mini-column. In Figure 2, *J*_*ee*_, *J*_*ie*_, *J*_*ii*_, *J*_*ei*_, and *J*^*external*^_*ie*_ are the weights of neuron connections. These coefficients indicate the strength of synaptic connections of the excitatory neurons to each other, the synaptic strength of the excitatory neurons to the inhibitory neurons in the same mini-column, the strength of synaptic connections of the inhibitory neurons to each other, the synaptic strength of the inhibitory neurons to the excitatory neurons, and the synaptic strength of the excitatory neurons to the inhibitory neurons in the adjacent mini-column, respectively.

Each excitatory pool receives two synaptic currents: one from its own neurons and the other from the neurons of the inhibitory pool in the same mini-column (see Figure 2). Equation (5) shows these currents. In this equation, *V_*ee*_* and *V_*ei*_* are equal to 0 and −80, respectively.

(5) $$I_{syne}^{\left(1\right)}=J_{ee}g_{e}^{\left(1\right)}\left(V_{ee}-V_{e}\left[j\right]\right)+J_{ei}g_{i}^{\left(1\right)}\left(V_{ei}-V_{e}\left[j\right]\right)$$

Each inhibitory pool receives three synaptic currents as described in Equation (6). One synaptic current is exerted from the excitatory pool in the same mini-column, the second one is from its own neurons, and the last one is from the excitatory pool of the adjacent mini-column.

(6) $$I_{syni}^{\left(1\right)}=J_{ie}g_{e}^{\left(1\right)}\left(V_{ie}-V_{i}\left[j\right]\right)+J_{ii}g_{i}^{\left(1\right)}\left(V_{ii}-V_{i}\left[j\right]\right)\;+\;J_{ie}^{external}g_{e}^{\left(2\right)}\left(V_{ie}-V_{i}\left[j\right]\right)$$

In Equation (6) *V_*ie*_* is 0 and *V_*ii*_* is −80. Equation (5) is added to Equation (1a) for excitatory pools and Equation (6) is added to Equation (1a) for inhibitory pools. As *V_*ii*_* and *V_*ei*_* are considered less than the minimum value of the action potential of a neuron, the corresponding synaptic current is always negative, so it is always opposed to increase the voltage value. Thus, this can be an expression of the inhibitory synaptic current.

The total effect of the synaptic variable of the excitatory and inhibitory pools on post-synaptic neurons is shown by *g*_*X*_, *X* ∈ {*e*, *i*} in Equation (7). Based on all-to-all connections in each pool, *g*_*X*_ is equal for all neurons in the same pool and it is obtained by the average of pre-synaptic neurons effects in each pool. In Equation (7), *N_*X*_* is the number of neurons in each pool.

(7) $$g_{X}=\frac{\sum_{k\;=\;1}^{N_{X}}\;s_{X}[k]}{N_{X}}$$

According to Börgers et al. (2005), *s_*X*_*[*k*] is the gating variable, which is calculated by the following equation. τ_R_ and τ_D_ for AMPA receivers are equal to 0.2, 2 and for GABA_A_ receivers are equal to 0.5, 10, respectively.

(8) $$\frac{ds}{dt}=\frac{1+\tan\text{h}\left(\frac{V}{10}\right)}{2}\frac{1-s}{\tau_{R}}-\frac{s}{\tau_{D}}$$

The data transmission rate of neurotransmitters between the pre-synaptic and post-synaptic neurons is modeled by the mechanism of synapse. The term [1 + tanh(V/10)]/2 can be assumed as a normalized neurotransmitter concentration (Börgers et al., 2005).

### FPGA implementation

In this section, the FPGA implementation of the neural network, shown in Figure 2, is described. According to Figure 2, this network is made of two similar mini-columns, each has two neural pools: excitatory and inhibitory. The neurons of the excitatory and inhibitory pools have the same structure and there is no significant difference in their synaptic mechanisms. The selected single neuron model for implementation is described completely in Bonabi et al. (2012a) and we made a few changes in the model in this work. We reduced the order of dynamics of the system to make it simpler for the implementation of big neural networks. As Equations (1a–4c) describe, the dynamics of *m* and *h* are neglected. The most difficult part in the implementation of equations is the implementation of the exponential function. The accuracy and performance in the implementation of the exponential function has an intensive impact on the results. We use hyperbolic Coordinate Rotation Digital Computer (CORDIC) algorithm, which could be implemented using simple shifters and adders, to calculate the function. Equations (9a–c) shows the CORDIC algorithm used in our implementation (Ercegovac and Lang, 2003).

(9a) $$x\left[j+1\right]=x\left[j\right]+\sigma_{j}2^{-j}y\left[j\right]$$

(9b) $$y\left[j+1\right]=y\left[j\right]+\sigma_{j}2^{-j}x\left[j\right]$$

(9c) $$z\left[j+1\right]=z\left[j\right]-\sigma_{j}tanh^{-1}(2^{-j})$$

In order to minimize the required FPGA resources, we use LUT for implementing hyperbolic tangent inverse function in the Equation (9c). Therefore, we achieve less running time, because only a simple search is required instead of a mathematical calculation. CORDIC algorithm can calculate the value of functions with a reasonable error only when the inputs are in a limited range (Ercegovac and Lang, 2003). According to Equation (10), in order to have a bigger range of inputs, we separate each input into two parts: integer part and fractional part. We precalculate the exponential of the integer part using MATLAB and save them in a LUT. The exponential of the fractional part is calculated using the implemented CORDIC module. Then, in order to produce the final value of the exponential function, the output of LUT is multiplied by the output of the implemented CORDIC module.

(10) A=B+C⇒exp(A)=exp(B) · exp(C)

Another module needed to produce new values of *n*, *s*, and *V* from their dynamics is the integrator. The method used to implement this module is the same as our previous work (Bonabi et al., 2012a). Equation (11) is used to implement the integrator.

(11) $$x\left(t+\Delta t\right)=x\left(t\right)+\Delta t.\dot{x}(t)$$

**Figure 3:**
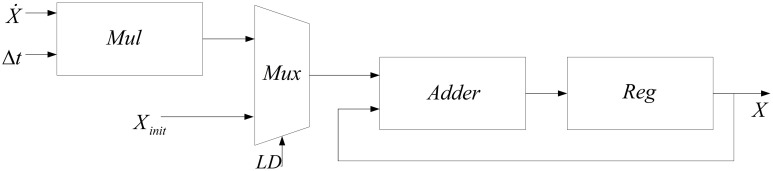
**Block diagram of implemented module for calculating the integral function**.

In this work, we add a multiplexer (Mux) in order to insert the initial values for the dynamics *n*, *s*, *V*. Figure 3 shows the block diagram of the implemented module for calculating the integral function. In order to calculate the integral of each step, a register is used to save the previous values of the integral.

**Figure 4:**
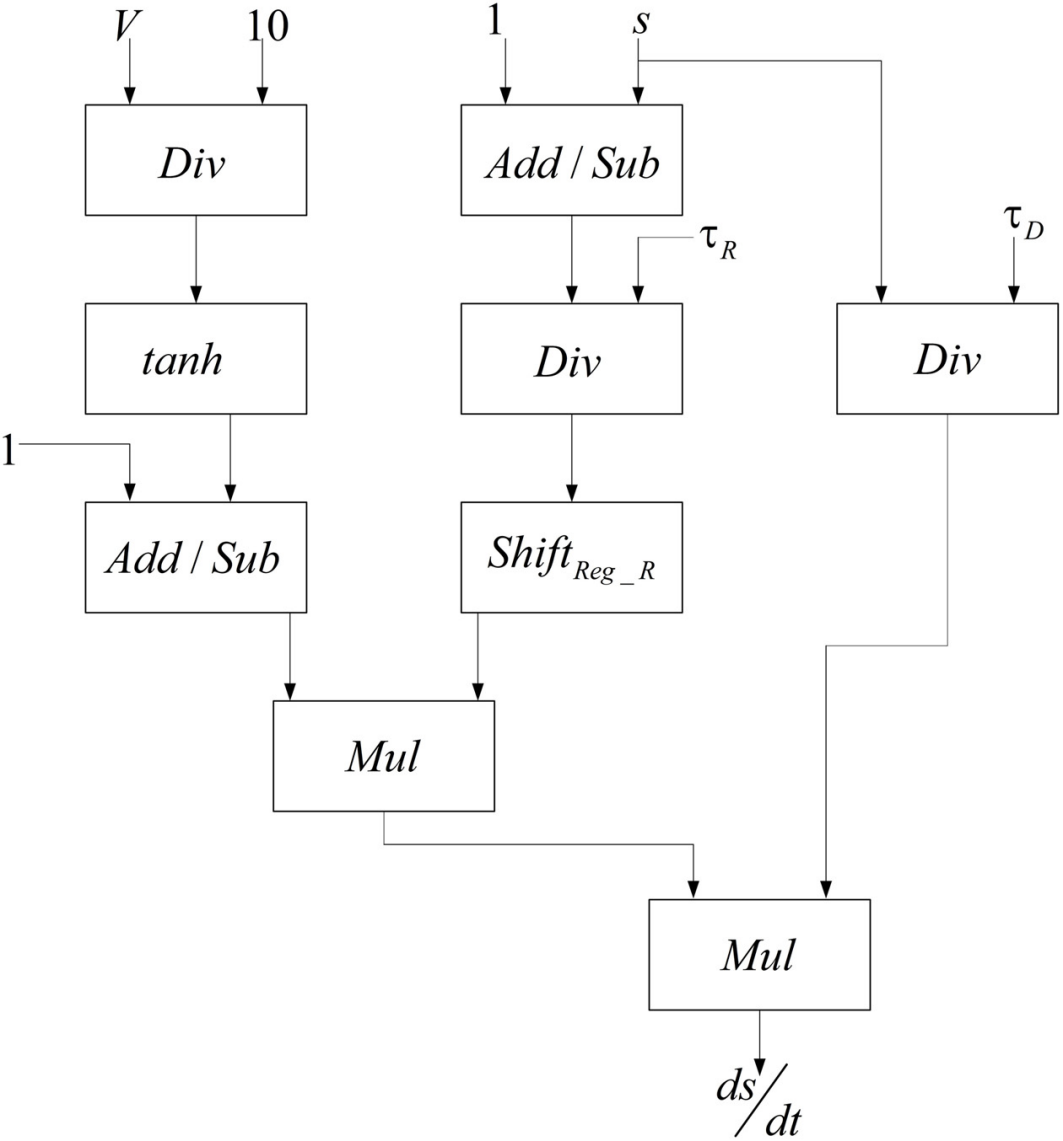
**Designed module to implement the gating variable *s***.

The basis of implementations of the excitatory and the inhibitory pools are the same and it is similar to the method presented in Bonabi et al. (2012b). We only change the number of neurons and the number of synaptic currents in each pool. Neurons are connected to each other with neurotransmitters, which make synaptic current. Synaptic current, as shown in Equations (5, 6), depends on the total effects of the synaptic variable and the gating variable. Therefore, for calculating synaptic current, we have to implement a module that could calculate the gating variable. The block diagram in Figure 4 is used to implement the gating variable, *s*, which is shown in Equation (8).

In order to calculate the hyperbolic tangent block in Figure 4, we use Equation (12) by employing our implemented exponential function.

(12) $$tanh\left(x\right)=\frac{exp\left(2x\right)-1}{\exp\left(2x\right)+1}$$

**Figure 5:**
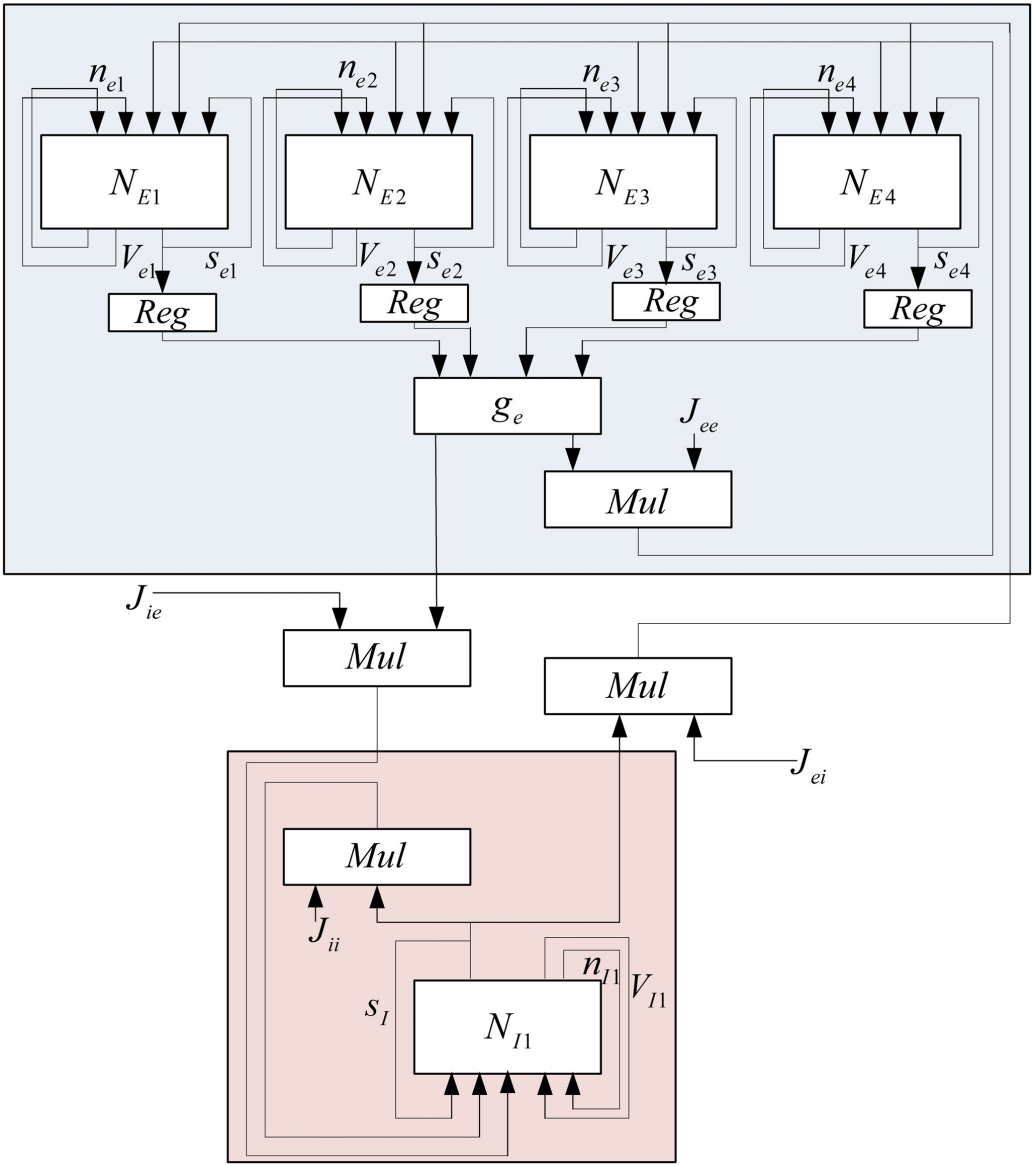
**Block diagram of implemented mini-column with five neurons**.

To reduce the number of multipliers, a one-bit left shifter is used to duplicate the input of hyperbolic tangent. These currents are added to the implemented pools in order to make a connection between the neurons in each pool, between excitatory and inhibitory pools in each mini-column, and excitatory and inhibitory pools in the different mini-columns. Because of the limited usable area, the implemented mini-column has five neurons, four of them are in excitatory pool, and the last one is in inhibitory pool. The block diagram of this neural pool is shown in Figure 5.

**Figure 6:**
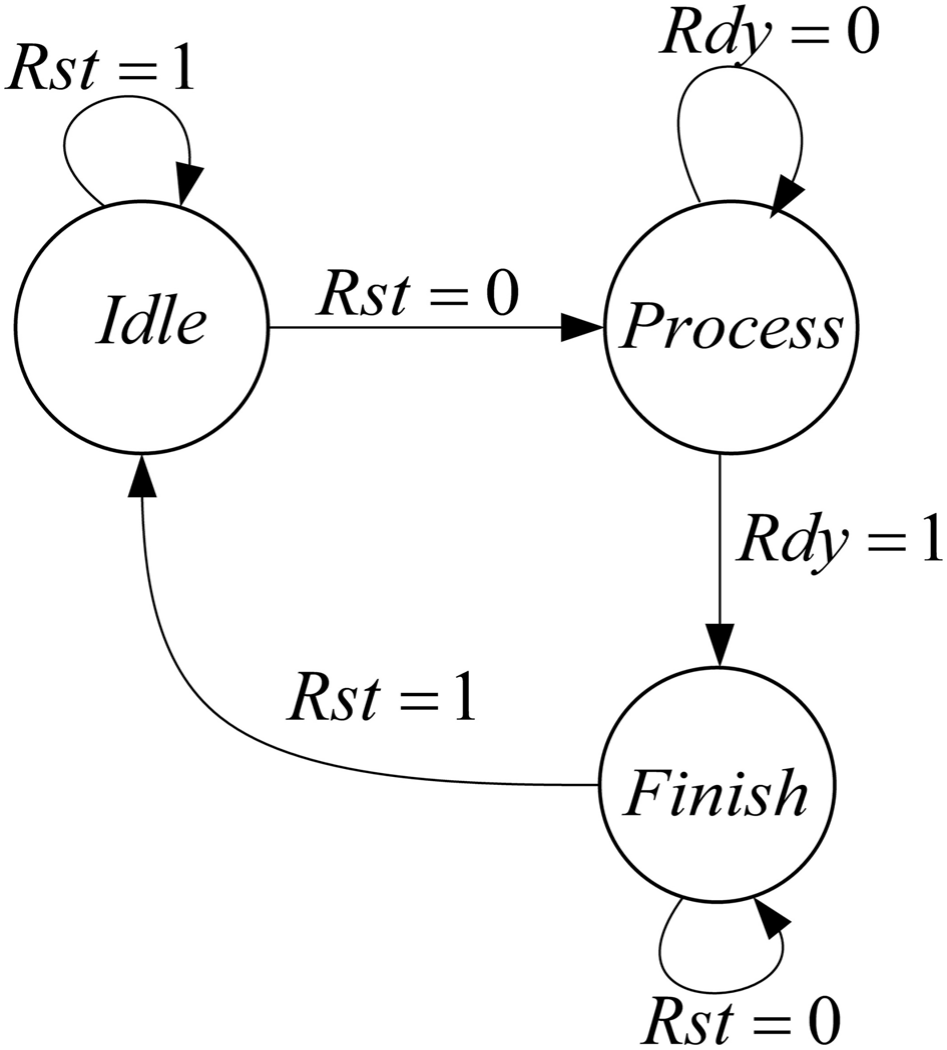
**Designed controller for the implemented mini-column**.

Figure 6 shows the controller that is designed for this mini-column. This mini-column finishes its work when both of the pools finish their processing.

**Figure 7:**
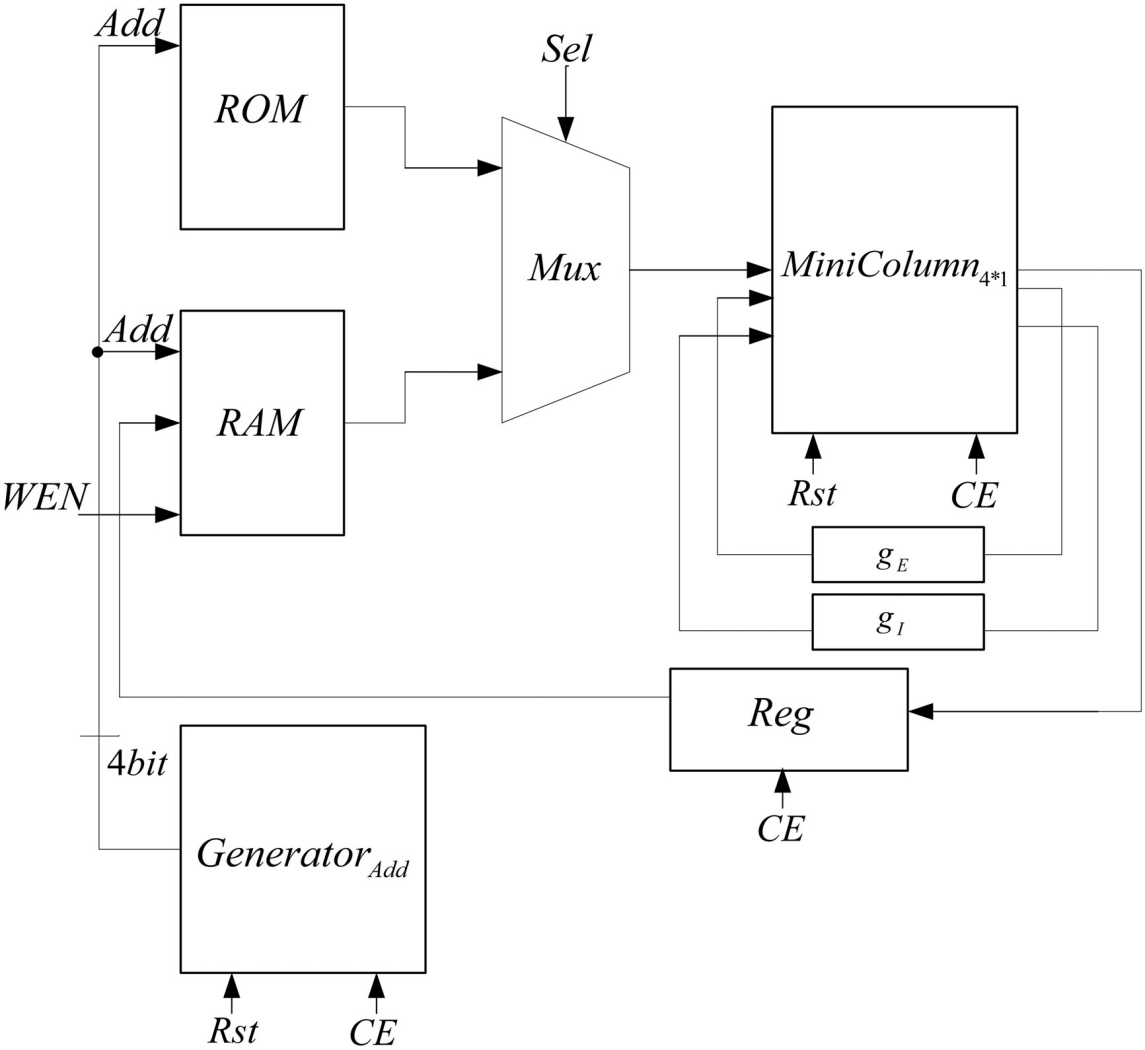
**The proposed architecture to increase the number of neurons in the mini-column**.

To implement the neural network with 150 neurons, two mini-columns with 75 neurons are needed which are connected to each other by a synaptic current. Because of the resource limitation of FPGA, we share resources by multiplexing in time to implement this neural network on a single chip. Figure 7 shows the architecture that is used to increase the number of neurons in each mini-column. In this architecture, we use a ROM to put the initial values of membrane voltages and gating variables for each of the neurons. The RAM is used to write the responses of the neurons and use them for the next executions of the system. Both of the ROM and RAM have 15 rows; each row has data for all of the five neurons in the mini-column. Each row is used in each execution. The Mux is used to choose the data from ROM and RAM. The address generator generates the address to read from the ROM/RAM and write in the RAM.

**Figure 8:**
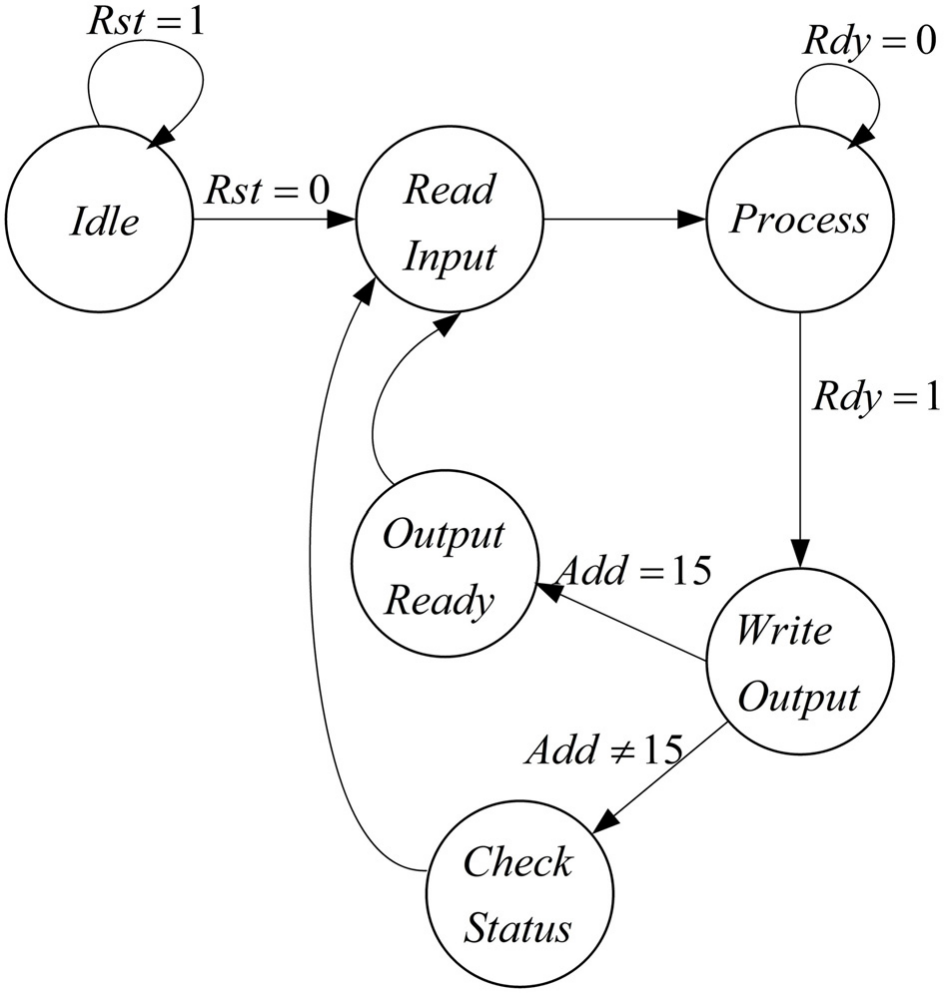
**The designed controller for the implemented architecture to increase number of neuron in the mini-column**.

The proposed architecture has a specific controller, shown in Figure 8, which controls all the processes. We have a counter in this controller, which manages the interactions of all components. The proposed architecture and the controller work in these steps:

- At first, the controller resets the circuit in order to set the values of all components to zero and the controller moves to the next state.
- In the second state (Read Input) the select input of Mux is set to zero and the inputs of the mini-column is read from the first line of the ROM. For the next 15 executions of this system, the select input of Mux is zero to choose the data from ROM. After first 15 executions, the select input is changed to one and data will be read from RAM. After reading the inputs of mini-column, the controller moves to the next state.
- The controller will stay in this state (Process) until the responses of neurons (five neurons in the mini-column) are ready. When the ready output of the mini-column is activated, the state of controller changes to the next state (Write Output).
- In the Write Output state, the answer of five neurons (membrane voltages and gating variables) is written in the same row of the RAM that data was read from it. If the address reaches to 15, the controller changes its state to Output Ready state; otherwise, the next state will be Check Status. Then, after a clock cycle, the state of the controller will be changed to Read Input, address generator points to the next row of the ROM/RAM, and these steps will be repeated.
- If the value of the address generator reaches 15, the controller changes its state to Output Ready. In this state, the ready output of mini-column with 75 neurons is activated. Then, all of the synaptic currents will be calculated and given to the pools. The controller resets the counter in the address generator, so address generator will point to the first row of the RAM and these steps will be repeated until the desired final time.

**Figure 9:**
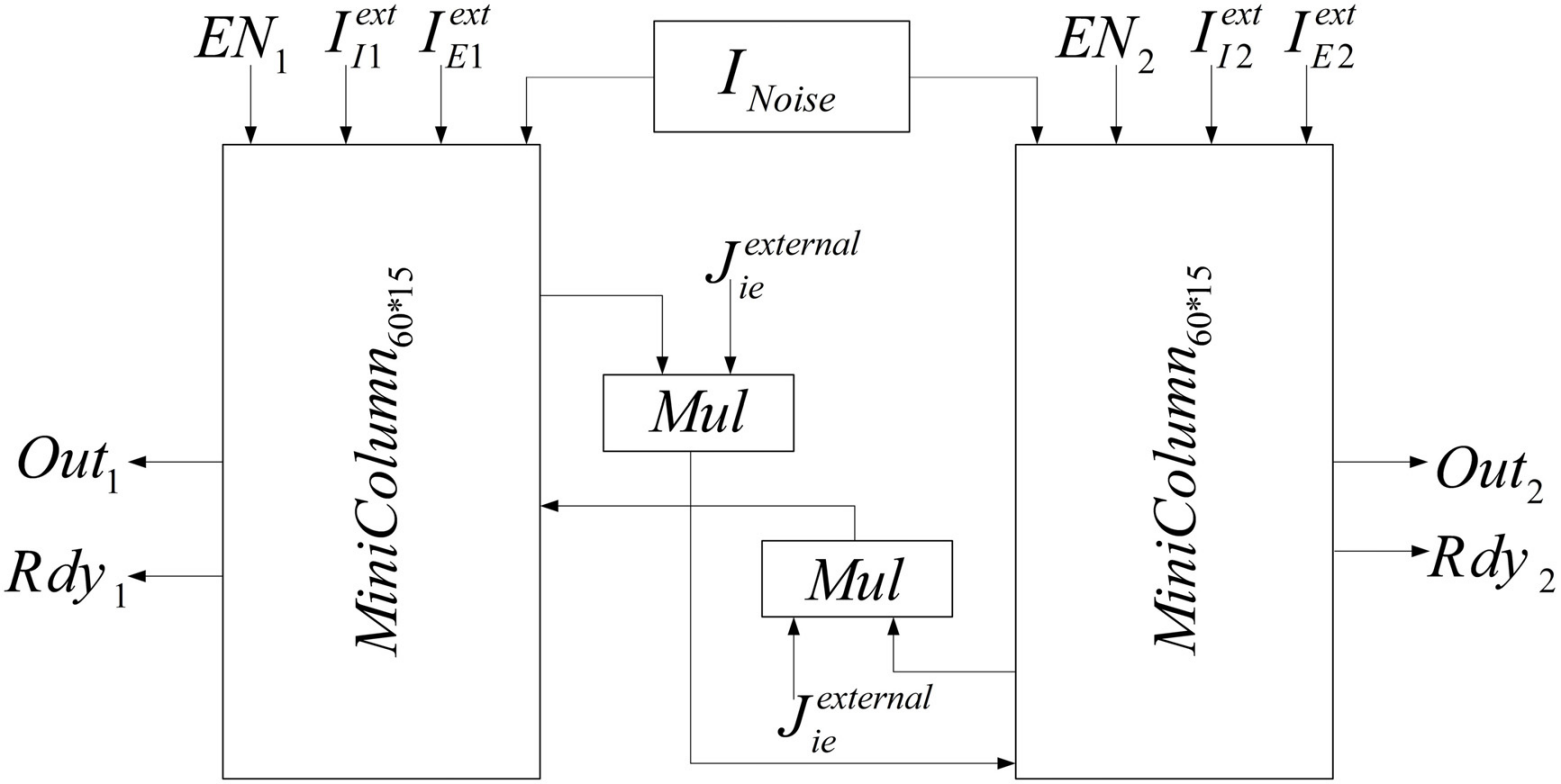
**Block diagram of implemented neural network**.

In the neural network, two mini-columns should be connected to each other by synaptic currents. To implement this connection when *g*_*X*_ is calculated in each mini-column, it will be entered to the inhibitory pool of the adjacent mini-column by coefficient of *J*^*external*^_*ie*_ and the synaptic current will be calculated in the inhibitory pools. The block diagram of the implemented neural network is shown in Figure 9.

**Figure 10:**
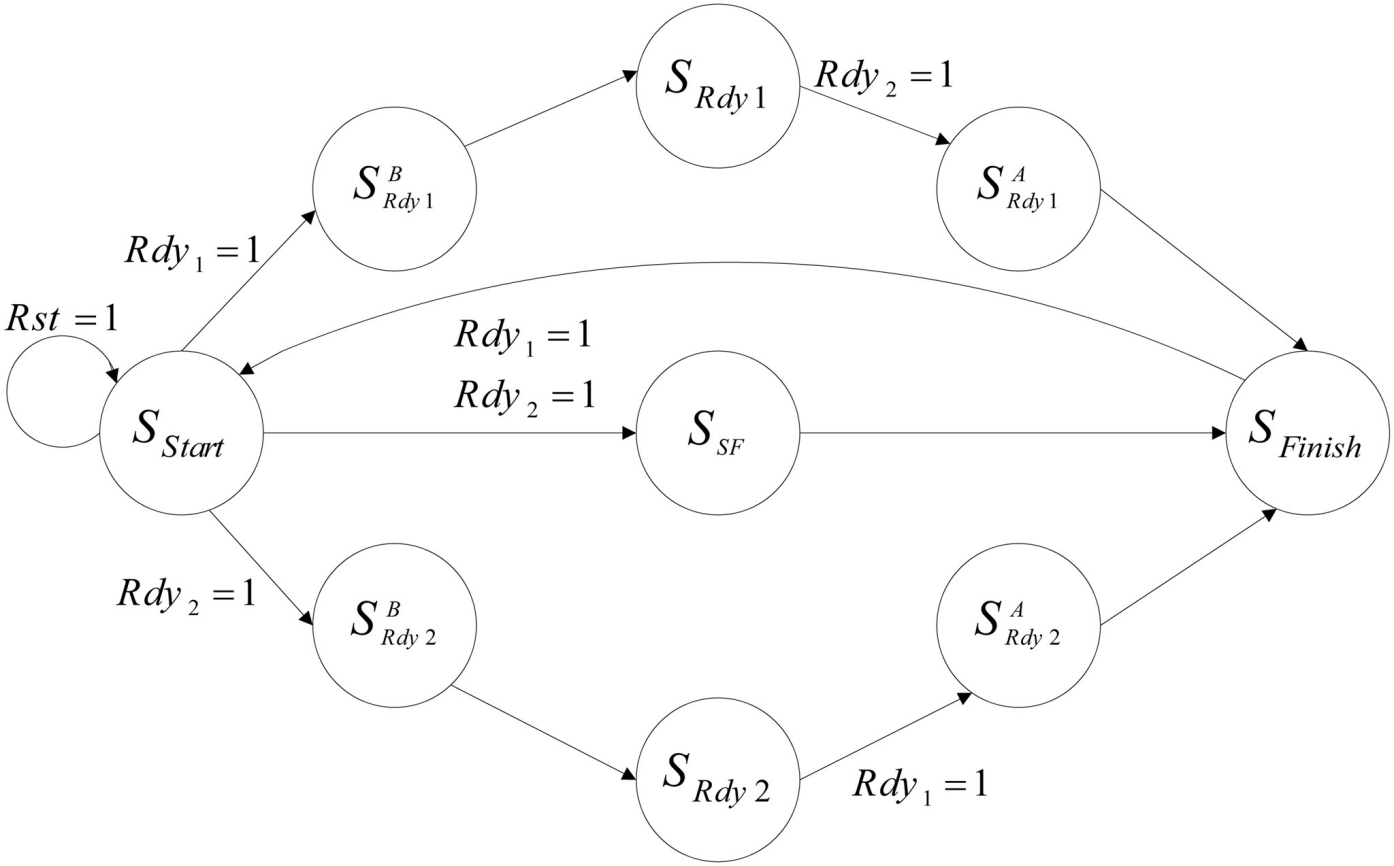
**The designed controller, which is used to control the neural network**.

Figure 10 shows the designed controller for the implemented neural network. At first, the controller resets both of the mini-columns and they start to work from the S_Start_ state. Then, mini-columns are activated together and work as a parallel system until both of them prepare the response of their 75 neurons. Each mini-column that finishes working, activates its ready output (Rdy). According to the ready output of the mini-columns, the controller changes its state. For example, if the ready output of the first mini-column is activated (Rdy_1_ = 1), the controller changes its state to S^B^_*Rdy*1_. In the new state, the clock of this mini-column is deactivated. Then, the controller changes its state to the next state (S_Rdy1_). State of the controller would not change until the responses of all of the neurons in the other mini-column are prepared. Then, the controller moves to the next state (S^A^_*Rdy*1_), the clock pulse of both of the mini-columns are deactivated in this state, and after a clock cycle the controller moves to the final state (S_Finish_). In the final state, the results are written in the memories of the mini-columns (RAM) and the controller changes its state to the first state (S_Start_). The controller repeats this process until the desired time.

In order to add the effect of stochastic factors, we add a noise term to the input current of each neuron. This random term creates small differences between neurons, which makes the implementation more biologically plausible. The noises are generated from a zero mean Gaussian distribution for each neuron. Then, they are saved in a ROM that is shown in Figure 9 (*I_*Noise*_*). Each given address to the ROM by the address generator gives noises to the neurons of the two mini-columns.

## Results

To validate the implemented neural network on FPGA, the deployed bit level simulations are compared with the numerical implementations of the mathematical models in MATLAB Simulink.

All of the hardware components are designed and implemented using VHDL modeling language. The main objective in the design of the low-level implementation is the accuracy of the output. Due to the hardware constraints, in the implementation of all modules minimum number of bits are used. More details of implementation are given in Table 1 as synthesis results.

**Table 1:** **Abstract of synthesis results**.

**Criteria**	**Virtex-7**	
	**xq7k410t-2I-rf676**	
	**Used**	**Utilization (%)**
Frequency	63.386 MHz	–
No. of LUTs	86032	33
No.of LUT-FF pairs	30528	28
No.of slice registers	50228	9
No.of DSP blocks	1112	72
No.of BRAM	14	1

**Figure 11:**
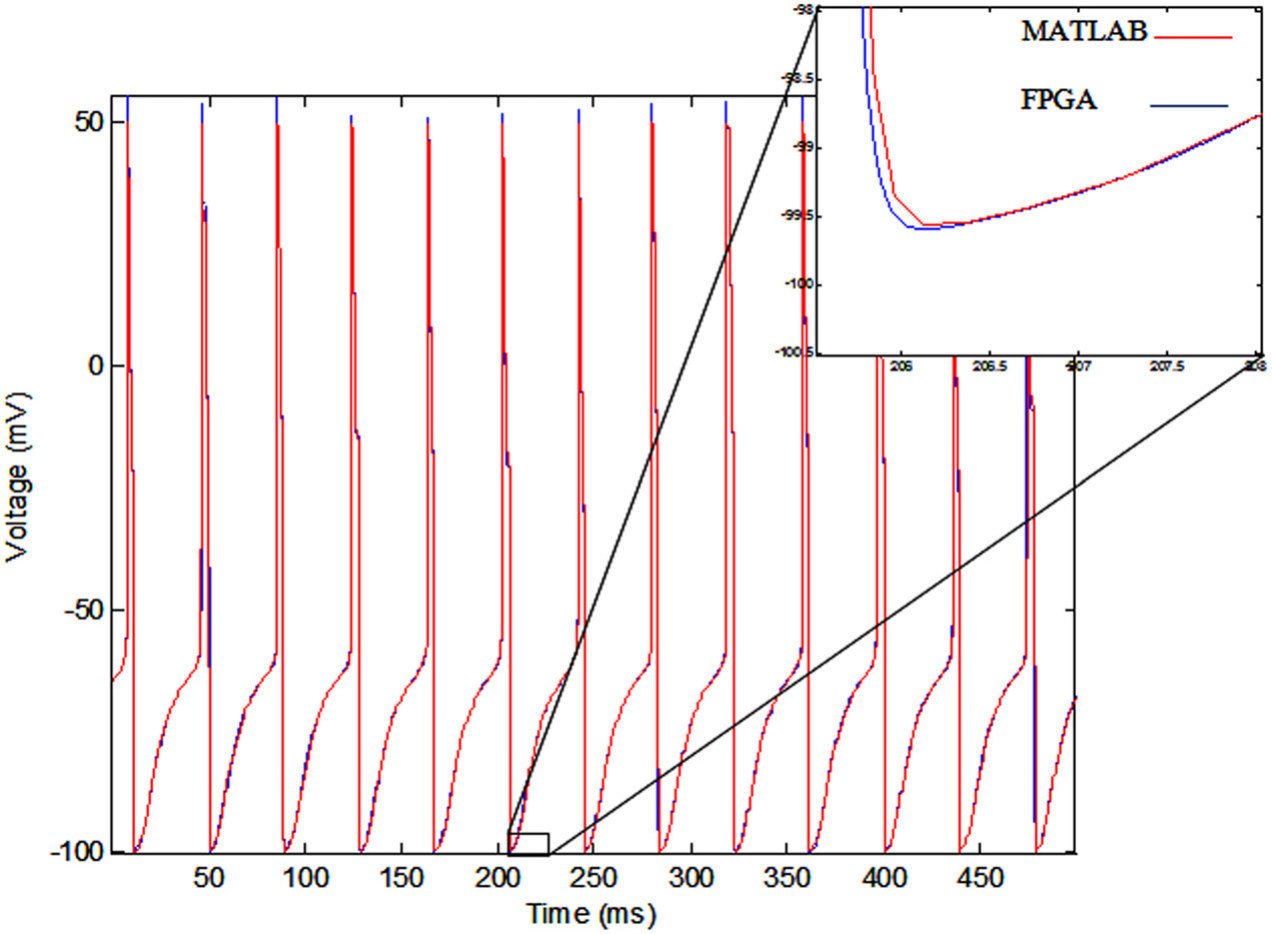
**The voltage of single neurons, implemented single neuron (blue line) and MATLAB simulation (red line)**.

The membrane voltage of the implemented single neuron (blue line) and MATLAB simulation (red line) for *I*_*ext*_ = 0.7 mA are shown in Figure 11. According to this figure, there is a small difference between the membrane voltages of the implemented model on FPGA and MATLAB simulation, because of the rounding error in the digital implementation. However, the firing rates of both of the signals are equal.

In an excitatory pool (when there is no connection between the pools of a mini-column) each neuron receives an excitatory synaptic current and external stimulus. Therefore, a neuron in an excitatory pool receives two exciting current while a free single neuron receives just an external stimulus. Thus, the firing rate of a neuron in an excitatory pool is higher than a free single neuron. Each neuron in the inhibitory pool receives an inhibitory synaptic current that reduces the effect of external stimulus. Therefore, the firing rate of a neuron in the inhibitory pool would be less than the firing rate of a free single neuron. In order to investigate the effects of synaptic currents in the pools, we exerted a certain stimulation (*I*_*ext*_ = 0.7 mA) to a free single neuron, an excitatory pool, and an inhibitory pool. The result shows that in the excitatory pool the firing rate is more than the firing rate of the free single neuron and in the inhibitory pool it is less than the firing rate of the free single neuron. The firing rate in the single neuron, excitatory pool, and inhibitory pool are shown in the Table 2.

**Table 2:** **Firing rates of a neuron in single neuron and pools**.

	**Single neuron**	**Excitatory pool**	**Inhibitory pool**
Firing rate (HZ)	26	30	16

**Figure 12:**
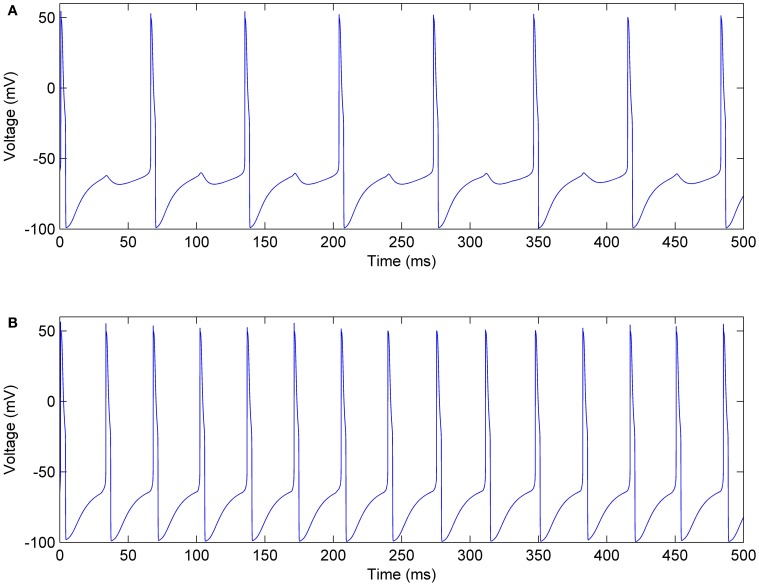
**Membrane voltage of one of the neurons, (A) In the excitatory pool, (B) In the inhibitory pool of a mini-column**.

In each mini-column, the excitatory pool receives an inhibitory current from the inhibitory pool that reduces the firing rates of the neurons. Figure 12 shows the membrane voltages of one neuron in the excitatory and inhibitory pools, given *I*_*E*_ = 0.8 mA, *I*_*I*_ = 0.7 mA, *J*_*ie*_ = 0.7, and *J*_*ei*_ = 0.2. According to the voltages in this figure, when the neurons in the inhibitory pool reach their maximum value, they prevent the neurons in the excitatory pool to complete their firing. Therefore, as you can see in Figure 12A small bumps are created in the shape of the voltages of the excitatory neurons. In addition, the firing rate of the neurons in the excitatory pools is reduced.

**Figure 13:**
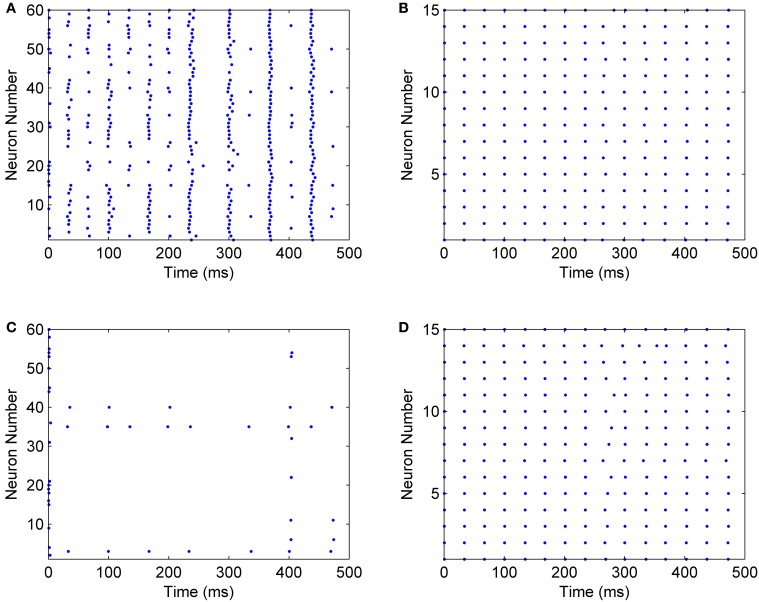
**Rastergrams of the implemented neural network, for (A) The excitatory pool in mini-column 2, (B) The inhibitory pool in mini-column 2, (C) The excitatory pool in mini-column 1, (D) The inhibitory pool in mini-column 1**.

When mini-columns are connected to each other, and a stimulation excites one of the excitatory pools in the network, both of the inhibitory pools will be activated and they try to inhibit their excitatory pools. For example, consider a case when the excitatory pool in the second mini-column (*E*2) receives greater stimulation than the excitatory pool in the first mini-column (*E*1). *E*2 and *E*1 exert excitatory synaptic currents to both of the second and first inhibitory pools (*I*2 and *I*1 respectively) (see Figure 2). *I*1 and *I*2 try to suppress the effect of the stimulations to *E*1 and *E*2. Since *E*1 is less stimulated than *E*2, the inhibiting effect of *I*1 might be able to suppress the neural activity in *E*1. To investigate this on the implemented system, we injected the same currents to both of the inhibitory pools and *E*1 is stimulated with less external current than *E*2. As rastergrams in Figure 13 shows, the neurons in *E*1 are strongly suppressed by *I*1, while *I*2 is not able to suppress *E*2. Rastergrams of *I*1 and *I*2 looks almost the same, because both of them are excited by *E*1 and *E*2 and they receive equal external stimulations. The external currents and weights of neuron connections are as follow:

$$\begin{array}{l} I_{E}^{\left(2\right)}=0.85mA,I_{I}^{\left(2\right)}=0.7mA,I_{E}^{\left(1\right)}=0.7mA,I_{I}^{\left(1\right)}=0.7mA\\ J_{ee}=0.1,J_{ie}=0.7,J_{ii}=0.02,J_{ei}=0.2,J_{ie}^{external}=0.45. \end{array}$$

## Discussion

There is a range of neuron models: from abstract to biophysically plausible ones (Bailey et al., 2011). The computational efficiency of models is inversely proportional to inclusion of details in them. Therefore, simpler models are more widely used. Abstract neuron models are usually used in the artificial neural network implementations; see Khan et al. (2006); Sahin et al. (2006); Çavuşlu et al. (2011) as examples. Interest in implementation of spiking neuron models is also increased in the recent years (Yang et al., 2011; Ambroise et al., 2013).

Several implementations of biologically plausible neuron models and neural networks have also been proposed. For example, in Zou et al. (2006), the authors have implemented a neural ınetwork based on the H-H neuron model using analog ASIC and a computer. In another example, in Heo and Song (2012) a VLSI implementation of a biological neuron model is presented. Each specific ıimplementation has its own advantages over any general-purpose implementation, like realization on FPGA. Nevertheless, the inherent parallel processing nature of FPGA devices, in addition to their shorter design time and online re-configurability, make them a good alternative for implementation of biologically plausible neural networks.

There are FPGA implementations of simple neuron models; see Chen and Wang (2002) and Yang et al. (2011) for Integrate-and-Fire and Leaky Integrate-and-Fire models respectively. In Bailey et al. (2011) Cellular Automata is implemented on FPGA. In Cassidy and Andreou (2008); Mokhtar et al. (2008); Rice et al. (2009); Ambroise et al. (2013) the Izhikevich model, which is a compromise between biological and abstract neuron models, has been implemented on FPGA. We are interested to have a more biologically plausible neuron model; so the H-H neuron model is chosen. There are different FPGA implementations of the H-H neuron model (Graas et al., 2004; Zhang et al., 2009; Pourhaj et al., 2010; Saïghi et al., 2010a,b; Grassia et al., 2011). A LUT-based implementation of computing algorithms is used in Graas et al. (2004), that has better time complexity, but more area is needed compared to the direct implementation. In addition, it has lower accuracy than direct algorithmic implementation. Therefore, a large size of memory is used to save the pre-computed results with limited number of bits, which reduces the final accuracy because of the limitations on memory size. Moreover, in Graas et al. (2004) MATLAB System Generator (SG) is used to implement a neuron and it is obvious that SG cannot produce an optimal hardware. Pourhaj et al. (2010) used LUT-based implementation in different parts of the model and some equations with the exponential terms, which has a ıside effect on the final accuracy of implementation. The authors of Zhang et al. (2009) implemented a 32-bit floating point reconfigurable somatic neuroprocessor on an FPGA. In Saïghi et al. (2010a,b); Grassia et al. (2011) the H-H based neural network has been implemented on a combination of systems: digital hardware, analog hardware, and software. Due to data transmission between different interfaces, these kinds of implementations are more sensitive to noise and have lower speed compared to the network implemented on a single FPGA. Also, implementation, validation, and evaluation of these types of systems are harder than pure digital design on FPGA devises. In Bonabi et al. (2012b), a neural pool is implemented on FPGA. In this paper, we have upgraded this pool to a network composed of four different pools. It is done by improving our implementation method through using computational techniques, such as CORDIC algorithm and step-by-step integration in the implementation of arithmetic circuits, in addition to sharing resources. Our implementation makes increasing the network size possible while keeping the network execution speed close to real time and having high precision.

## Conclusions and future work

In this paper, we introduced a method to implement a biologically plausible neural network on an FPGA. The network consists of four neural pools; two excitatory; and two inhibitory pools. Each pool is implemented based on the H-H neuron model.

We used MATLAB simulation for validating our implementation. After simulating the network using MATLAB, we chose suitable number of bits for each variable in mathematical computations, in order to have less error in the results of the implemented network.

We described each part of the H-H model equations using VHDL as a hardware description language (Bottom-up approach). In order to have a neural network with the maximum number of neurons and accurate responses, we focused on having small error, rapid processing time, and using less hardware resources. In the implementation, both of the full implementation and LUT-based implementation were used to obtain high accuracy and low execution time. In addition, varieties of computational circuits were tested to have an implementation with optimal resource usage, such as CORDIC algorithm and step-by-step integrator, which are beneficial in having accurate response and low hardware resource usage. At every stages of the implementation, range of the inputs and outputs are examined, in order to achieve the appropriate data representation and to have simplified arithmetic functions. For example, in the implementation of different modules, the LUT implementation is used instead of complete functional implementation, where the function has limited range of changes in the values of inputs and outputs. We achieved high-speed performance of running the neural network, based on the parallel processing nature of FPGA, which is practically impossible in sequential platforms. Moreover, our design has high accuracy in its output; i.e., in membrane voltage signals and firing rates.

The presented implementation technique has other benefits as well, such as scalability and extendibility. The number of neurons could be increased by increasing the number of repetitions in the address generator. It results in linear increase in response time of the system. The implemented network could be used as a pipeline system to raise its speed in the next steps of this research. Using our system for developing controllers for cognitive robots is among our research plans.

### Conflict of interest statement

The authors declare that the research was conducted in the absence of any commercial or financial relationships that could be construed as a potential conflict of interest.
